# Addressing the problem of obesity and associated cardiometabolic risk in black South African women – time for action!

**DOI:** 10.1080/16549716.2017.1366165

**Published:** 2017-10-10

**Authors:** Julia H. Goedecke

**Affiliations:** ^a^ Non-Communicable Disease Research Unit, South African Medical Research Council, Cape Town, South Africa

**Keywords:** Obesity, metabolic syndrome, black African women, physical activity, sedentary behaviour, adiponectin, cardiometabolic fitness, body size perceptions, lifestyle interventions

## Abstract

The PhD thesis of Gradidge, entitled ‘Factors associated with obesity and metabolic syndrome in an ageing cohort of black women living in Soweto, Johannesburg (Study of Women in and Entering Endocrine Transition [SWEET])’, attempts to understand the determinants of obesity and metabolic syndrome (MetS) in a population of urban-dwelling black South African women. A conceptual framework is presented, which positions obesity as the central risk factor for MetS, and includes the possible influence of socioeconomic status, lifestyle behaviours and body size perceptions, as key determinants of obesity. This commentary focuses on the two main findings of Gradidge’s thesis, namely, (i) physical activity and sedentary behaviour, and (ii) body composition and adiponectin, as risk factors for obesity and MetS in black South African women. Despite a high prevalence of obesity (48%), Gradidge showed that 75% of the women taking part in the study were meeting WHO guidelines on physical activity. This commentary suggests that the relationship between physical activity and cardiometabolic risk may be confounded by socioeconomic status. Alternatively, the intensity, and not necessarily the volume, of activity, as well as high rates of sedentary behaviour are posited as important determinants of obesity and MetS in black South African women. Accordingly, this commentary questions the veracity of the WHO guidelines on physical activity in developing countries, where most women meet the guidelines but have very poor cardiorespiratory fitness, are obese and are at high risk of MetS. Gradidge also showed that the most consistent and significant correlate of MetS in this cohort of middle-aged women was low serum levels of adiponectin. This commentary highlights various lifestyle interventions that have been shown to increase adiponectin levels. Finally, the importance of immediate action to address the problem of obesity and MetS is emphasised.

## The problem of obesity and cardiometabolic risk in South African women

The thesis of Gradidge attempts to understand the determinants of obesity and metabolic syndrome (MetS) in a population of urban-dwelling black South African women [1]. This is particularly pertinent, given that black South African women have the highest prevalence of obesity (42%) within sub-Saharan Africa [2], which is associated with a high prevalence of MetS (42%) [3] and type 2 diabetes (14.7%) [4]. As South Africa is further along the epidemiological transition than other countries in sub-Saharan Africa, knowledge of the determinants of obesity and MetS gained from this thesis may be useful for intervention development in the broader sub-Saharan African context.

In his thesis, Gradidge presents an excellent conceptual framework, which positions obesity as the central risk factor for MetS, and includes the possible influence of socioeconomic status, lifestyle behaviours and body size perceptions, as key determinants of obesity [1]. Gradidge’s thesis tests this hypothesis through secondary analyses of 10-year prospective data from the caregivers of the Birth-to-Twenty longitudinal cohort study [5]. Over this 10 year follow-up period, the prevalence of obesity increased by 29–66% [6], while the prevalence of MetS in this same cohort at baseline was as high as 50%. Notably, 74% of the women underestimated their actual body sizes [6]; this is consistent with similar research within South Africa [7] and sub-Saharan Africa [8], confirming that African women have a greater body size tolerance, and highlighting the challenges associated with weight-loss interventions in sub-Saharan Africa.

In this commentary, I will focus on the two main findings of this PhD thesis, namely, (i) physical activity and sedentary behaviour, and (ii) body composition and adiponectin, as risk factors for obesity and MetS in black South African women.

## Physical activity and sedentary behaviour as risk factors for obesity and MetS

Despite a high prevalence of obesity (48%), Gradidge showed that 75% of the women were meeting the WHO physical activity guidelines [9] (accumulating 150 min of moderate intensity activity per week or equivalent), confirming the results of similar studies in South Africa [10,11] and sub-Saharan Africa [12]. Further, he showed that meeting physical activity guidelines was not associated with reduced obesity or body fat, or improved cardiometabolic outcomes. Rather greater physical activity was associated with lower socioeconomic status, and, in particular, not having a motor vehicle or TV. These findings suggest that the relationship between physical activity and cardiometabolic risk may be confounded by socioeconomic status. Although Gradidge did measure socioeconomic status based on the ownership of household commodities, other measures of socioeconomic status, including income, sanitation, food security and access to health care, were not measured; this should be considered in understanding these associations. Notably, age, socioeconomic status (ownership of commodities), body composition and measures of physical activity accounted for only 1–13% of the variance in obesity or cardiometabolic risk [13], highlighting the need to gain a more in-depth understanding of the determinants of obesity and MetS in this population.

An alternative suggestion for the paradox of high levels of physical activity in combination with high rates of obesity and MetS may relate to the intensity of the physical activity undertaken. Most physical activity was accrued by walking for travel, which is done at a low-to-moderate intensity, suggesting that the intensity, and not necessarily the volume of activity, may be the important determinant of obesity and MetS. Indeed, Gradidge showed that the most consistent determinant of change in body fat over the 10-year follow-up period was vigorous physical activity. However, very few women actually performed vigorous activity, even in the group meeting physical activity guidelines, with a median of 0 min (interquartile range: 0–0 min) vigorous activity for both active and inactive groups [13]. Dickie et al. [14], using objectively measured physical activity in a younger cohort of black South African women, showed that no women participated in vigorous physical activity, and that when engaged in physical activity, most time was spent in light activity. Further, Dickie et al. [14] showed that cardiorespiratory fitness, rather than light or moderate physical activity, was associated with reduced adiposity and lower cardiometabolic risk. The estimated average maximal oxygen consumption (VO_2max_) of these women was ‘very poor’, based on guidelines from the American College of Sports Medicine, and fell below the 5th percentile for women of a similar age range.

Gradidge also showed that the women included in his study were sedentary (estimated by self-reported time spent in sitting) for 3 h per day, which is substantially lower than that measured objectively in the study of Dickie et al. [14], who showed that the majority of time (59%) during waking hours was spent in sedentary behaviours (±8.5 h/day). Nonetheless, both studies showed that sedentary time was associated with increased concentrations of serum triglyceride. Accordingly, Gradidge’s solution to addressing the high rates of obesity in African women is to target sitting time by increasing time spent on unstructured movement or standing breaks, and reducing TV time. Notably, Dickie et al. [14] showed an inverse correlation between sitting time and cardiorespiratory fitness, such that targeting sedentary behaviour might also have an impact on cardiorespiratory fitness and vice versa.

The results of this thesis and related sub-Saharan Africa studies therefore question the veracity of the WHO guidelines on physical activity in developing countries where most women are meeting the physical activity guidelines, but have very poor cardiorespiratory fitness, are obese and at high risk of MetS. Rather, the focus should be on increasing cardiorespiratory fitness and reducing sedentary behaviour, while still maintaining high levels of physical activity. As most activity is accumulated from walking for travel, women should be encouraged to increase the intensity of this activity. Alternatively, other leisure activities (sports, gym, aerobics, etc.), which are typically performed at a higher intensity than transport- or work-related activities, might be suggested as alternative interventions to increase cardiorespiratory fitness. However, barriers to leisure activities, including lack of time and facilities, safety issues and negative community perceptions regarding weight loss [15] need to be addressed before interventions can be implemented.

## Adiponectin and body composition as risk factors for obesity and MetS

The most consistent and significant correlate of MetS in this cohort of middle-aged women was a low level of serum adiponectin. Adiponectin is an adipocyte-derived hormone that has insulin-sensitising and anti-inflammatory effects. Low adiponectin levels have been implicated in the pathogenesis of obesity-linked disorders, such as insulin resistance, dyslipidaemia, coronary heart disease, hypertension and type 2 diabetes [16]. Although identified as a major risk factor for MetS in Gradidge’s thesis, the potential to manipulate adiponectin levels, and hence reduce the risk of MetS was not considered. Numerous studies have considered this. For example, the *Look AHEAD* study showed that an intensive lifestyle intervention increased adiponectin levels, and was associated with improvements in fitness and weight loss in overweight or obese women with type 2 diabetes [17]. Similarly, the *POUNDS Lost* trial, which included a sample of overweight or obese adults, found that long-term (2 years) weight-loss dietary interventions varying in macronutrient composition increased levels of circulating adiponectin, and were associated with reductions in central adiposity and improvements in serum concentrations of lipids, independent of weight change [18]. Further, Wang et al. [19] showed that the addition of aerobic exercise training to an energy-restricted dietary intervention increased adiponectin concentrations, largely as a result of increased adiponectin release from abdominal and gluteal subcutaneous adipose tissue. Additionally, various dietary interventions, including adopting a Mediterranean diet, supplementation with omega-3 fatty acids, and even administration of garlic extract, have been shown to increase adiponectin levels [20].

Gradidge also showed an inverse association between abdominal subcutaneous adipose tissue (measured using ultrasound) and MetS, which is supported by a number of studies [21,22]. Apart from the metabolic sink hypothesis that he presents, the ‘protective’ nature of subcutaneous adipose tissue may be related to the depot differences in adiponectin secretion, with subcutaneous adipose tissue exhibiting greater adiponectin secretion than visceral adipose tissue, even in states of adipose tissue dysfunction, including obesity [23].

Gradidge highlights a novel finding in his thesis, showing that greater truncal fat-free soft tissue mass was associated with MetS; more specifically, low concentrations of high-density lipoproteins. This finding is difficult to reconcile, given that fat-free soft tissue mass of the trunk incorporates everything that is not bone or fat, and therefore includes muscle and organs. Although it will be helpful to examine this further using MRI, as he suggested, I believe that this is a type 1 error, which resulted from entering multiple collinear variables in a regression model and using backwards stepwise removal of non-significant variables.

## Conclusions

In his thesis, Gradidge highlights that although most women are sufficiently active, according to WHO recommendations, there is a large and increasing problem of obesity and MetS in black South African women. Although Gradidge showed that 74% of the women involved in the study underestimated their body size, just over half (57.4%) of the women expressed a desire to be leaner. This demonstrates a shift in mindset of black South African women who traditionally regarded obesity as a sign of wealth, respect, happiness and not having HIV [7], and emphasises that the time to intervene is now. Lifestyle interventions that focus on reducing energy intake, improving dietary quality and increasing cardiometabolic fitness, have been shown to be successful for weight loss, increasing adiponectin and reducing MetS. However, in a country where more than 50% of the population experience some form of food insecurity [24], and experience numerous barriers to exercise, this poses a major challenge. Nonetheless, all South Africans, from the individuals themselves to government, need to take up this challenge to prevent this epidemic from crippling the country.

## Paper context

This is a commentary on the PhD thesis of Gradidge, which attempts to understand the determinants of obesity and metabolic syndrome (MetS) in a population of urban-dwelling black South African women. The commentary focuses on the two main findings of this thesis, namely, (i) physical activity and sedentary behaviour, and (ii) body composition and adiponectin, as risk factors for obesity and MetS in black South African women.
